# Prevalence of coronary risk factors in contemporary practice among patients undergoing their first percutaneous coronary intervention: Implications for primary prevention

**DOI:** 10.1371/journal.pone.0250801

**Published:** 2021-06-09

**Authors:** Zoya Gurm, Milan Seth, Edouard Daher, Elizabeth Pielsticker, M. Imran Qureshi, Mark Zainea, Michael Tucciarone, George Hanzel, Peter K. Henke, Devraj Sukul

**Affiliations:** 1 Wayne State University School of Medicine, Detroit, MI, United States of America; 2 University of Michigan Health System, Ann Arbor, MI, United States of America; 3 Ascension St. John Hospital, Detroit, MI, United States of America; 4 Henry Ford Allegiance Health, Jackson, MI, United States of America; 5 Detroit Medical Center-Sinai Grace Hospital, Detroit, MI, United States of America; 6 McLaren Macomb Hospital, Mount Clemens, MI, United States of America; 7 Beaumont Hospital, Troy, MI, United States of America; 8 Emory University Structural Heart and Valve Center, Atlanta, GA, United States of America; Hospital Clinico San Carlos, SPAIN

## Abstract

**Background:**

Cigarette smoking, hypertension, dyslipidemia, diabetes, and obesity are conventional risk factors (RFs) for coronary artery disease (CAD). Population trends for these RFs have varied in recent decades. Consequently, the risk factor profile for patients presenting with a new diagnosis of CAD in contemporary practice remains unknown.

**Objectives:**

To examine the prevalence of RFs and their temporal trends among patients without a history of myocardial infarction or revascularization who underwent their first percutaneous coronary intervention (PCI).

**Methods:**

We examined the prevalence and temporal trends of RFs among patients without a history of prior myocardial infarction, PCI, or coronary artery bypass graft surgery who underwent PCI at 47 non-federal hospitals in Michigan between 1/1/2010 and 3/31/2018.

**Results:**

Of 69,571 men and 38,930 women in the study cohort, 95.5% of patients had 1 or more RFs and nearly half (55.2% of women and 48.7% of men) had ≥3 RFs. The gap in the mean age at the time of presentation between men and women narrowed as the number of RFs increased with a gap of 6 years among those with 2 RFs to <1 year among those with 5 RFs. Compared with patients without a current/recent history of smoking, those with a current/recent history of smoking presented a decade earlier (age 56.8 versus 66.9 years; p <0.0001). Compared with patients without obesity, patients with obesity presented 4.0 years earlier (age 61.4 years versus 65.4 years; p <0.0001).

**Conclusions:**

Modifiable RFs are widely prevalent among patients undergoing their first PCI. Smoking and obesity are associated with an earlier age of presentation. Population-level interventions aimed at preventing obesity and smoking could significantly delay the onset of CAD and the need for PCI.

## Introduction

Coronary artery disease (CAD) is the leading cause of death globally [1]. Epidemiologic studies have established cigarette smoking, hypertension, dyslipidemia, diabetes, and obesity as conventional risk factors for CAD. Progress in preventing, identifying, and controlling these risk factors has contributed to the decreased incidence of cardiac disease [2]. For instance, there has been a general decline in smoking in the United States [3]. The prevalence of high total cholesterol has also declined [4]. Additionally, hypertension control has improved, although a large proportion of the population, including younger and minority patients continue to have poorly controlled hypertension [5]. After increasing in the 1980s and 1990s, the prevalence of obesity has remained stable in the 2000s [6]. Alternatively, the incidence of diabetes has generally increased among adults, with obesity as a contributing factor [7].

While the importance of these five risk factors has been well-established, in the context of these opposing trends in their prevalence, the risk factor profile for patients presenting with new onset coronary artery disease (CAD) in the contemporary era remains unknown. A better understanding of the risk profile of patients with CAD in contemporary practice can help target primary prevention efforts. Accordingly, we examined the prevalence of risk factors in patients without known prior CAD who presented for their first percutaneous coronary intervention (PCI) in Michigan using the Blue Cross Blue Shield of Michigan cardiovascular collaborative (BMC2) clinical PCI registry. Additionally, we evaluated temporal trends in these risk factors over the past decade among these patients.

## Methods

All patient data points were derived from a HIPAA-compliant database. The University of Michigan IRB has waived the need for ongoing IRB approval on all analysis that are performed using BMC2 data. Consent was not obtained as all data were analyzed anonymously.

### Study population

Our study population included patients presenting for their first PCI between January 1, 2010 and March 31, 2018. The BMC2 clinical PCI registry has been previously described in detail [8]. Briefly, BMC2 is a quality improvement collaborative of all non-federal hospitals that perform PCI in the state of Michigan that has been working for over 20 years to facilitate inter-institutional quality improvement. The authors are unable to share the raw data, due to contractual agreements between participating institutions and the BMC2 registry that prohibit data sharing with external agencies. However, the analysis code and metadata to support the study figures is available on request from Annemarie Forrest, Program Manager of the BMC2 (avassalo@med.umich.edu). To examine patients undergoing their first percutaneous coronary revascularization procedure, we excluded patients with a history of prior myocardial infarction (MI), PCI, or coronary artery bypass graft surgery (CABG). Traditional risk factors for CAD including hypertension, dyslipidemia, obesity, diabetes, and smoking were defined per the NCDR CathPCI Registry version 4.4 data dictionary [9]. Hypertension was defined as either having a history of hypertension diagnosed and treated with medication, diet, and/or exercise, having prior documentation of blood pressure greater than 140 mm Hg systolic and/or 90 mm Hg diastolic for patients without diabetes or chronic kidney disease, prior documentation of blood pressure greater than 130 mm Hg systolic and/or 80 mm Hg diastolic on at least two occasions for patients with diabetes or chronic kidney disease, or as being currently on pharmacologic therapy for treatment of hypertension. Dyslipidemia was defined as having a total cholesterol greater than 200 mg/dL (5.18 mmol/l), low-density lipoprotein greater than or equal to 130 mg/dL (3.37 mmol/l), or high density lipoprotein less than 40 mg/dL (1.04 mmol/l). Obesity was defined as having a BMI greater than 30. Presence of diabetes mellitus was defined as having been previously diagnosed by a physician to have diabetes mellitus or as having a fasting blood sugar greater than 126 mg/dL (7 mmol/L). Current or recent smoking was defined as use of cigarettes any time in the year prior to presentation.

### Statistical analysis

Age at presentation for patient cohorts defined by combinations of risk factors were presented graphically as means ± standard deviation, and differences in age between cohorts were assessed for statistical significance at alpha = 0.05 significance level using the Wilcoxon rank sum test. No adjustments were made for multiple comparisons.

## Results

During the study period, 251,142 PCI procedures were performed, of which 142,066 (56.6%) were excluded due to a history of prior PCI, AMI, or CABG surgery and 575 (0.23%) were excluded because of missing risk factor data. A total of 38,930 women and 69,571 men were included in our study population of patients without a prior history of revascularization or AMI who underwent PCI.

The risk factor profile and baseline characteristics of men and women are shown in **Table 1**. Among men, the most common risk factors were hypertension (71.9%) and dyslipidemia (65.1%), followed by obesity (44.9%), current/recent smoking (34.5%) and diabetes (27.8%). Among women, hypertension was present in 80.7%, dyslipidemia in 68.9%, obesity in 48%, diabetes in 35.5%, and 31% had a history of current/recent smoking. Nearly half of patients presented with 3 or more risk factors (55.2% of women and 48.7% of men), while only 4.5% of patients had none of the traditional risk factors documented (3.4% of women and 5.1% of men). The average number of risk factors was 2.51 (2.64 for women and 2.44 for men).

**Table 1:** Risk factor profile and baseline characteristics by age and gender.

	Total		≤ 45 years		46–55 years		56–65 years		66–75 years		> 75 years	
	women	men	women	men	women	men	women	men	women	men	women	men
**N:**	38,930	69,571	2,095	5,614	5,830	15,286	10,044	23,011	10,657	16,254	10,304	9,406
**% of total (by gender)**			5.38%	8.07%	14.98%	21.97%	25.80%	33.08%	27.37%	23.36%	26.47%	13.52%
**Individual CAD Risk Factors N(%)**												
Current Smoking	12,050 (31.0%)	23,978 (34.5%)	1,383 (66.0%)	3,488 (62.1%)	3,565 (61.1%)	8,017 (52.4%)	4,128 (41.1%)	8,456 (36.7%)	2,269 (21.3%)	3,229 (19.9%)	705 (6.8%)	788 (8.4%)
Hypertension	31,403 (80.7%)	50,020 (71.9%)	1,305 (62.3%)	3,169 (56.4%)	4,080 (70.0%)	9,755 (63.8%)	7,840 (78.1%)	16,369 (71.1%)	9,008 (84.5%)	12,859 (79.1%)	9,170 (89.0%)	7,868 (83.6%)
Hyperlipidemia	26,840 (68.9%)	45,264 (65.1%)	989 (47.2%)	2,816 (50.2%)	3,563 (61.1%)	9,046 (59.2%)	6,853 (68.2%)	15,099 (65.6%)	7,956 (74.7%)	11,706 (72.0%)	7,479 (72.6%)	6,597 (70.1%)
Diabetes	13,837 (35.5%)	19,334 (27.8%)	751 (35.8%)	1,150 (20.5%)	1,951 (33.5%)	3,623 (23.7%)	3,767 (37.5%)	6,432 (28.0%)	4,154 (39.0%)	5,297 (32.6%)	3,214 (31.2%)	2,832 (30.1%)
Obesity	18,687 (48.0%)	31,210 (44.9%)	1,306 (62.3%)	3,197 (56.9%)	3,236 (55.5%)	7,713 (50.5%)	5,463 (54.4%)	10,800 (46.9%)	5,308 (49.8%)	6,929 (42.6%)	3,374 (32.7%)	2,571 (27.3%)
***No*. *of Risk Factors***												
0	1,332 (3.4%)	3,555 (5.1%)	84 (4.0%)	226 (4.0%)	172 (3.0%)	670 (4.4%)	337 (3.4%)	1,199 (5.2%)	348 (3.3%)	857 (5.3%)	391 (3.8%)	603 (6.4%)
1	5,249 (13.5%)	12,126 (17.4%)	308 (14.7%)	1,131 (20.1%)	778 (13.3%)	2,911 (19.0%)	1,215 (12.1%)	3,909 (17.0%)	1,243 (11.7%)	2,483 (15.3%)	1,705 (16.5%)	1,692 (18.0%)
2	10,815 (27.8%)	19,924 (28.6%)	516 (24.6%)	1,550 (27.6%)	1,358 (23.3%)	3,994 (26.1%)	2,321 (23.1%)	6,214 (27.0%)	2,823 (26.5%)	4,696 (28.9%)	3,797 (36.8%)	3,470 (36.9%)
3	11,735 (30.1%)	20,138 (28.9%)	533 (25.4%)	1,481 (26.4%)	1,656 (28.4%)	4,307 (28.2%)	3,071 (30.6%)	6,721 (29.2%)	3,433 (32.2%)	5,035 (31.0%)	3,042 (29.5%)	2,594 (27.6%)
4	8,262 (21.2%)	11,722 (16.8%)	475 (22.7%)	984 (17.5%)	1,397 (24.0%)	2,686 (17.6%)	2,519 (25.1%)	4,184 (18.2%)	2,543 (23.9%)	2,875 (17.7%)	1,328 (12.9%)	993 (10.6%)
5	1,537 (3.9%)	2,106 (3.0%)	179 (8.5%)	242 (4.3%)	469 (8.0%)	718 (4.7%)	581 (5.8%)	784 (3.4%)	267 (2.5%)	308 (1.9%)	41 (.4%)	54 (.6%)
***Demographics & clinical characteristics***												
Race												
White	32,411 (83.3%)	60,736 (87.3%)	1,532 (73.1%)	4,586 (81.7%)	4,595 (78.8%)	13,188 (86.3%)	8,092 (80.6%)	20,131 (87.5%)	9,083 (85.2%)	14,361 (88.4%)	9,109 (88.4%)	8,470 (90.0%)
Black	5,501 (14.1%)	6,586 (9.5%)	517 (24.7%)	805 (14.3%)	1,107 (19.0%)	1,553 (10.2%)	1,678 (16.7%)	2,150 (9.3%)	1,264 (11.9%)	1,365 (8.4%)	935 (9.1%)	713 (7.6%)
Asian	348 (0.9%)	868 (1.2%)	14 (0.7%)	111 (2.0%)	34 (0.6%)	236 (1.5%)	88 (0.9%)	264 (1.1%)	112 (1.1%)	191 (1.2%)	100 (1.0%)	66 (0.7%)
American Indian or Alaskan Native	121 (0.3%)	211 (0.3%)	14 (0.7%)	22 (0.4%)	18 (0.3%)	54 (0.4%)	36 (0.4%)	69 (0.3%)	32 (0.3%)	49 (0.3%)	21 (0.2%)	17 (0.2%)
Native Hawaiian or Pacific Islander	32 (0.1%)	55 (0.1%)	1 (0.0%)	6 (0.1%)	6 (0.1%)	10 (0.1%)	6 (0.1%)	24 (0.1%)	12 (0.1%)	8 (0.0%)	7 (0.1%)	7 (0.1%)
Family History of Premature CAD	6,558 (16.8%)	11,378 (16.4%)	605 (28.9%)	1,599 (28.5%)	1,546 (26.5%)	3,589 (23.5%)	1,992 (19.8%)	3,839 (16.7%)	1,532 (14.4%)	1,779 (10.9%)	883 (8.6%)	572 (6.1%)
Prior Heart Failure	4,237 (10.9%)	4,789 (6.9%)	77 (3.7%)	163 (2.9%)	307 (5.3%)	543 (3.6%)	841 (8.4%)	1,239 (5.4%)	1,173 (11.0%)	1,434 (8.8%)	1,839 (17.8%)	1,410 (15.0%)
Currently on Dialysis	750 (1.9%)	1,036 (1.5%)	45 (2.1%)	73 (1.3%)	114 (2.0%)	172 (1.1%)	226 (2.3%)	336 (1.5%)	222 (2.1%)	270 (1.7%)	143 (1.4%)	185 (2.0%)
Cerebrovascular Disease	5,158 (13.2%)	5,773 (8.3%)	110 (5.3%)	127 (2.3%)	429 (7.4%)	611 (4.0%)	1,042 (10.4%)	1,530 (6.6%)	1,599 (15.0%)	1,820 (11.2%)	1,978 (19.2%)	1,685 (17.9%)
Peripheral Arterial Disease	3,902 (10.0%)	5,665 (8.1%)	68 (3.2%)	122 (2.2%)	389 (6.7%)	678 (4.4%)	892 (8.9%)	1,701 (7.4%)	1,265 (11.9%)	1,826 (11.2%)	1,288 (12.5%)	1,338 (14.2%)
Chronic Lung Disease	7,007 (18.0%)	8,896 (12.8%)	152 (7.3%)	227 (4.0%)	919 (15.8%)	1,256 (8.2%)	1,973 (19.6%)	2,905 (12.6%)	2,144 (20.1%)	2,703 (16.6%)	1,819 (17.7%)	1,805 (19.2%)
Diabetes Mellitus	13,837 (35.5%)	19,334 (27.8%)	751 (35.8%)	1,150 (20.5%)	1,951 (33.5%)	3,623 (23.7%)	3,767 (37.5%)	6,432 (28.0%)	4,154 (39.0%)	5,297 (32.6%)	3,214 (31.2%)	2,832 (30.1%)
CAD Presentation												
Asymptomatic, no angina	1,155 (3.0%)	2,475 (3.6%)	25 (1.2%)	64 (1.1%)	92 (1.6%)	315 (2.1%)	258 (2.6%)	773 (3.4%)	389 (3.7%)	831 (5.1%)	391 (3.8%)	492 (5.2%)
Symptom unlikely to be ischemic	1,162 (3.0%)	1,952 (2.8%)	17 (0.8%)	43 (0.8%)	105 (1.8%)	251 (1.6%)	246 (2.4%)	584 (2.5%)	388 (3.6%)	628 (3.9%)	406 (3.9%)	446 (4.7%)
Stable angina	3,752 (9.6%)	6,415 (9.2%)	73 (3.5%)	216 (3.8%)	398 (6.8%)	953 (6.2%)	984 (9.8%)	2,169 (9.4%)	1,331 (12.5%)	1,979 (12.2%)	966 (9.4%)	1,098 (11.7%)
Unstable angina	12,936 (33.2%)	21,169 (30.4%)	453 (21.6%)	1,132 (20.2%)	1,792 (30.7%)	4,150 (27.1%)	3,502 (34.9%)	7,233 (31.4%)	3,910 (36.7%)	5,616 (34.6%)	3,279 (31.8%)	3,038 (32.3%)
Non-STEMI	10,977 (28.2%)	18,126 (26.1%)	770 (36.8%)	1,820 (32.4%)	1,816 (31.1%)	4,321 (28.3%)	2,666 (26.5%)	5,745 (25.0%)	2,686 (25.2%)	3,725 (22.9%)	3,039 (29.5%)	2,515 (26.7%)
STEMI or equivalent	8,939 (23.0%)	19,423 (27.9%)	757 (36.1%)	2,338 (41.6%)	1,625 (27.9%)	5,295 (34.6%)	2,387 (23.8%)	6,503 (28.3%)	1,952 (18.3%)	3,470 (21.3%)	2,218 (21.5%)	1,817 (19.3%)

Abbreviations: CAD = coronary artery disease; STEMI = ST-elevation myocardial infarction.

Smoking and obesity tended to be more commonly observed in younger patients. Among patients ≤45 years old, 66.0% of women and 62.1% of men had a history of current/recent smoking. As age increased, the prevalence of smoking decreased to 61.1% in women and 52.4% in men aged 46–55 years, 41.1% in women and 36.7% in men aged 56–65 years, 21.3% in women and 19.9% in men aged 66–75 years, and was present in only 6.8% of women and 8.4% of men aged over 75 years. Similarly, obesity was prevalent in 62.3% of women and 56.9% of men ≤45 years of age, while it was prevalent in 32.7% of women and 27.3% of men >75 years old. Conversely, the prevalence of hypertension was higher in women and men >75 years old (89.0% and 83.6%) compared with patients ≤45 years old (62.3% and 56.4%).

Among 33,171 patients with diabetes, 2,269 (6.8%) were untreated prior to PCI admission, 2,049 (6.2%) were controlling diabetes through diet only, 16,396 (49.6%) were treated with oral agents without insulin, 12,205 (36.8%) were treated with insulin, and for 252 (0.8%) the treatment was unknown. Of 72,104 patients with dyslipidemia, information on statin use prior to PCI admission was available for 32,217 (44.7%). Of these patients, 20,723 (64.4%) had reported using a statin. The registry does not collect information on hypertension treatment prior to PCI admission.

Patients presenting with acute myocardial infarction (AMI) including ST-elevation and non-ST-elevation myocardial infarction had a higher prevalence of current/recent smoking compared with patients not presenting with AMI. Both AMI and non-AMI groups displayed the trend of patients who presented at a younger age having a higher prevalence of smoking and obesity compared with those who presented at older ages (**Table 2**).

**Table 2:** Prevalence of risk factors by age and gender among patients who underwent percutaneous coronary intervention for the treatment of acute myocardial infarction and non-acute myocardial infarction presentations.

**Acute myocardial infarction (STEMI and Non-STEMI)**										
	**≤ 45 years**		**46–55 years**		**56–65 years**		**66–75 years**		**> 75 years**	
	**Women**	**Men**	**Women**	**Men**	**Women**	**Men**	**Women**	**Men**	**Women**	**Men**
**N:**	1,534	4,184	3,464	9,687	5,083	12,338	4,667	7,249	5,287	4,361
**Individual Risk Factors**										
**Current/Recent Smoking**	1,084 (70.7%)	2,798 (66.9%)	2,369 (68.4%)	5,635 (58.2%)	2,532 (49.8%)	5,340 (43.3%)	1,301 (27.9%)	1,765 (24.3%)	423 (8.0%)	431 (9.9%)
**Hypertension**	866 (56.5%)	2,151 (51.4%)	2,182 (63.0%)	5,476 (56.5%)	3,631 (71.4%)	7,694 (62.4%)	3,656 (78.3%)	5,219 (72.0%)	4,564 (86.3%)	3,387 (77.7%)
**Hyperlipidemia**	619 (40.4%)	1,827 (43.7%)	1,799 (51.9%)	4,868 (50.3%)	2,928 (57.6%)	6,753 (54.7%)	3,057 (65.5%)	4,448 (61.4%)	3,449 (65.2%)	2,687 (61.6%)
**Diabetes**	478 (31.2%)	698 (16.7%)	958 (27.7%)	1,884 (19.4%)	1,652 (32.5%)	2,826 (22.9%)	1,667 (35.7%)	2,074 (28.6%)	1,599 (30.2%)	1,227 (28.1%)
**Obesity**	919 (59.9%)	2,304 (55.1%)	1,850 (53.4%)	4,610 (47.6%)	2,558 (50.3%)	5,277 (42.8%)	2,116 (45.3%)	2,807 (38.7%)	1,521 (28.8%)	1,065 (24.4%)
**Non-acute myocardial infarction**										
	**≤ 45 years**		**46–55 years**		**56–65 years**		**66–75 years**		**> 75 years**	
	**Women**	**Men**	**Women**	**Men**	**Women**	**Men**	**Women**	**Men**	**Women**	**Men**
**N:**	570	1,458	2,396	5,689	4,999	10,805	6,039	9,093	5,058	5,094
**Individual Risk Factors**										
**Current/Recent Smoking**	302 (53.0%)	702 (48.1%)	1,208 (50.4%)	2,422 (42.6%)	1,612 (32.2%)	3,158 (29.2%)	975 (16.1%)	1,477 (16.2%)	288 (5.7%)	358 (7.0%)
**Hypertension**	444 (77.9%)	1,029 (70.6%)	1,912 (79.8%)	4,325 (76.0%)	4,227 (84.6%)	8,745 (80.9%)	5,385 (89.2%)	7,701 (84.7%)	4,641 (91.8%)	4,506 (88.5%)
**Hyperlipidemia**	371 (65.1%)	999 (68.5%)	1,775 (74.1%)	4,210 (74.0%)	3,939 (78.8%)	8,404 (77.8%)	4,925 (81.6%)	7,296 (80.2%)	4,045 (80.0%)	3,931 (77.2%)
**Diabetes**	274 (48.1%)	455 (31.2%)	1,002 (41.8%)	1,755 (30.8%)	2,128 (42.6%)	3,632 (33.6%)	2,500 (41.4%)	3,246 (35.7%)	1,633 (32.3%)	1,618 (31.8%)
**Obesity**	389 (68.2%)	897 (61.5%)	1,389 (58.0%)	3,113 (54.7%)	2,907 (58.2%)	5,535 (51.2%)	3,202 (53.0%)	4,128 (45.4%)	1,858 (36.7%)	1,511 (29.7%)

Note: Percutaneous coronary intervention for the treatment of acute myocardial infarction was defined as undergoing percutaneous coronary intervention for ST-elevation or non-ST-elevation myocardial infarction presentations. All other presentations were considered non-acute myocardial infarction presentations.

**Fig 1:**
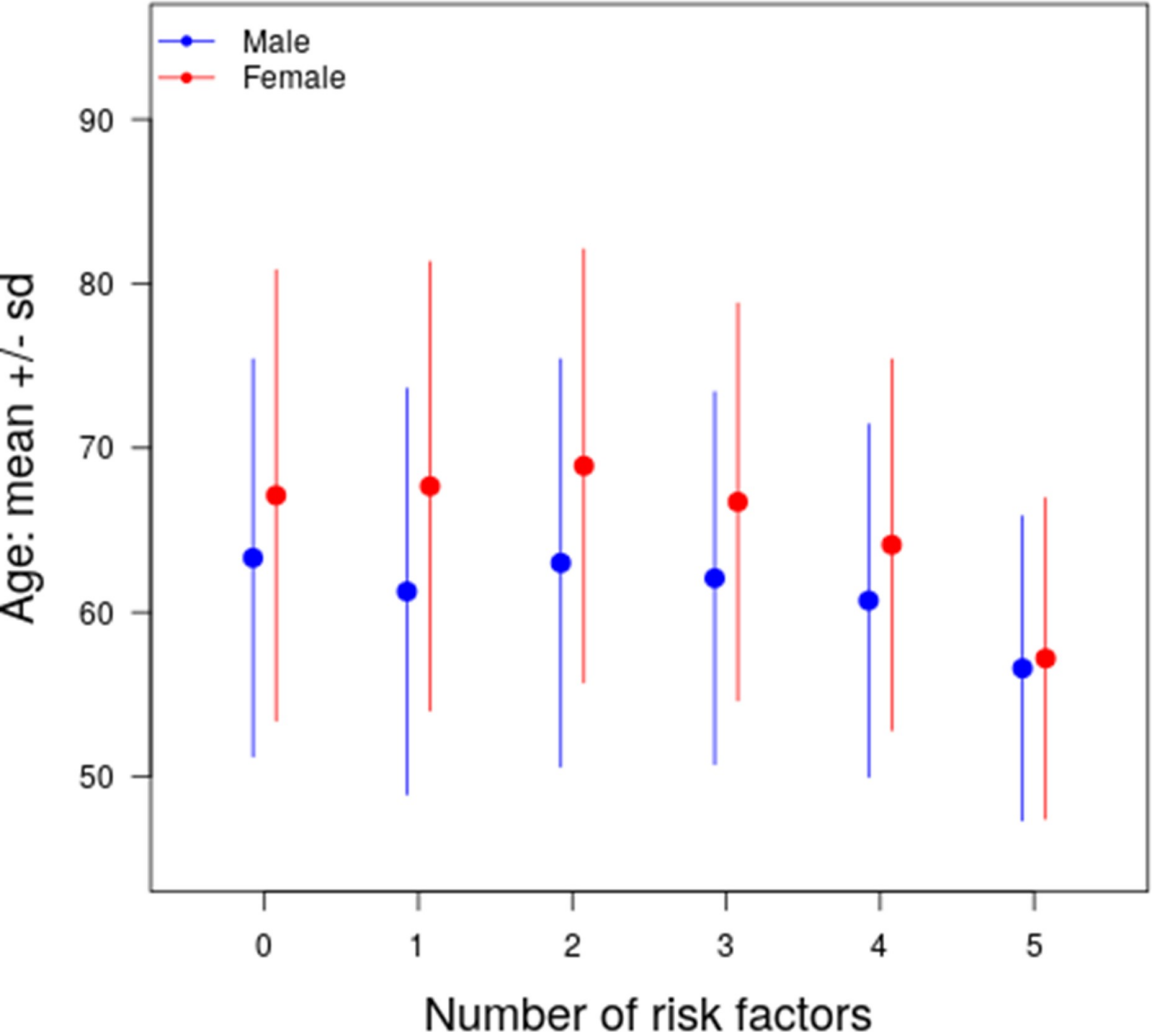
Age at the time of presentation for PCI by the number of risk factors stratified by gender. Risk factors include the presence of hypertension, diabetes, current/recent smoker, obesity, and dyslipidemia. Mean age (dot) and standard deviation (lines) of men and women are shown in blue and red, respectively.

**Fig 1** shows the mean age of presentation by number of CAD risk factors in men and women. Patients with none or one risk factor presented at a younger age than those with two or more traditional risk factors. As the number of risk factors increased from 2 to 5, the average age of presentation in each gender decreased from 68.9 +/- 13.2 for women and 63.0 +/- 12.4 for men with 2 risk factors to 57.2 +/- 9.8 for women and 56.6 +/- 9.3 for men with 5 risk factors. While women generally presented later than men, the gap in mean age between men and women narrowed as the number of risk factors increased from almost 6 years for patients with 2 risk factors to less than 1 year for patients with 5 risk factors.

**Fig 2:**
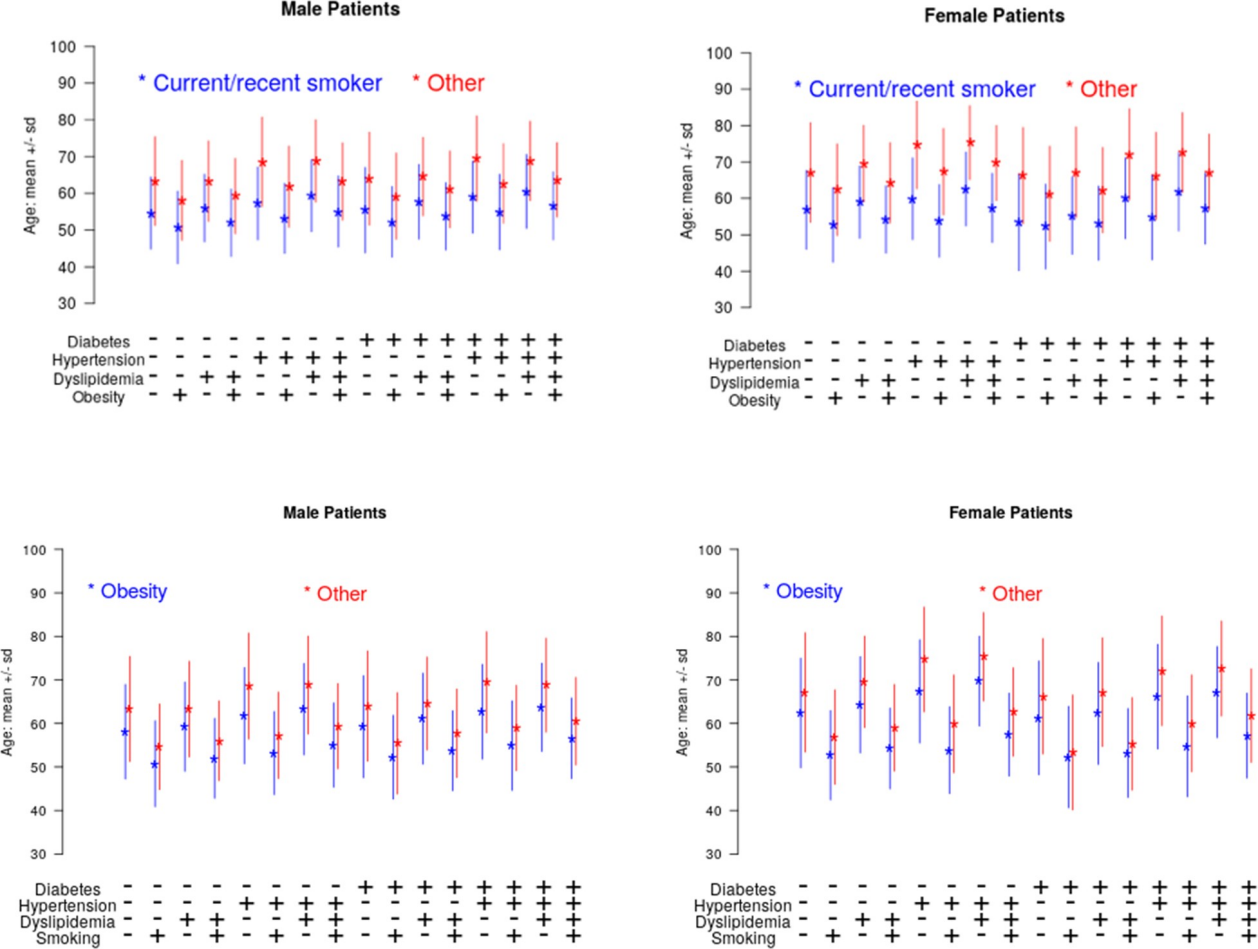
Age at the time of presentation by the presence and absence of a smoking and obesity across a background of other risk factors. Panel A depicts male patients smoking versus non-smoking. Panel B depicts female patients smoking versus non-smoking. Panel C depicts male patients who are obese vs non-obese. Panel D depicts female patients who are obese vs non-obese. Blue bars represent patients with the risk factor and red bars represent patients without the risk factor. Risk factor combinations are depicted along the x axis.

The association between smoking and obesity and patient age at the time of presentation is shown in **Fig 2**. Compared with those without a current/recent history of smoking, patients with a current/recent history of smoking presented nearly a decade earlier (age 56.8 versus 66.9 years, p < 0.0001). This association was seen in both men and women and across various combinations of risk factors (**Fig 2**). A similar trend was seen for obesity, where patients with obesity presented 4.0 years earlier (age 61.4 years versus 65.4 years, p < 0.0001). The average age at the time of presentation stratified by individual risk factors is shown in **Table 3**.

**Table 3:** Average age at the time of presentation among patients with and without individual risk factors.

	Patients without risk factor	Patients with risk factor	P-value	Difference in mean age
**Hypertension**	59.26 (11.99)	64.97 (12.10)	<0.001	5.71
**Diabetes**	63.15 (12.56)	64.42 (11.71)	<0.001	1.27
**Smoker**	66.90 (11.83)	56.79 (10.37)	<0.001	-10.11
**Obesity**	65.38 (12.60)	61.39 (11.63)	<0.001	-3.99
**Dyslipidemia**	61.31 (12.97)	64.67 (11.82)	<0.001	3.36

**Fig 3:**
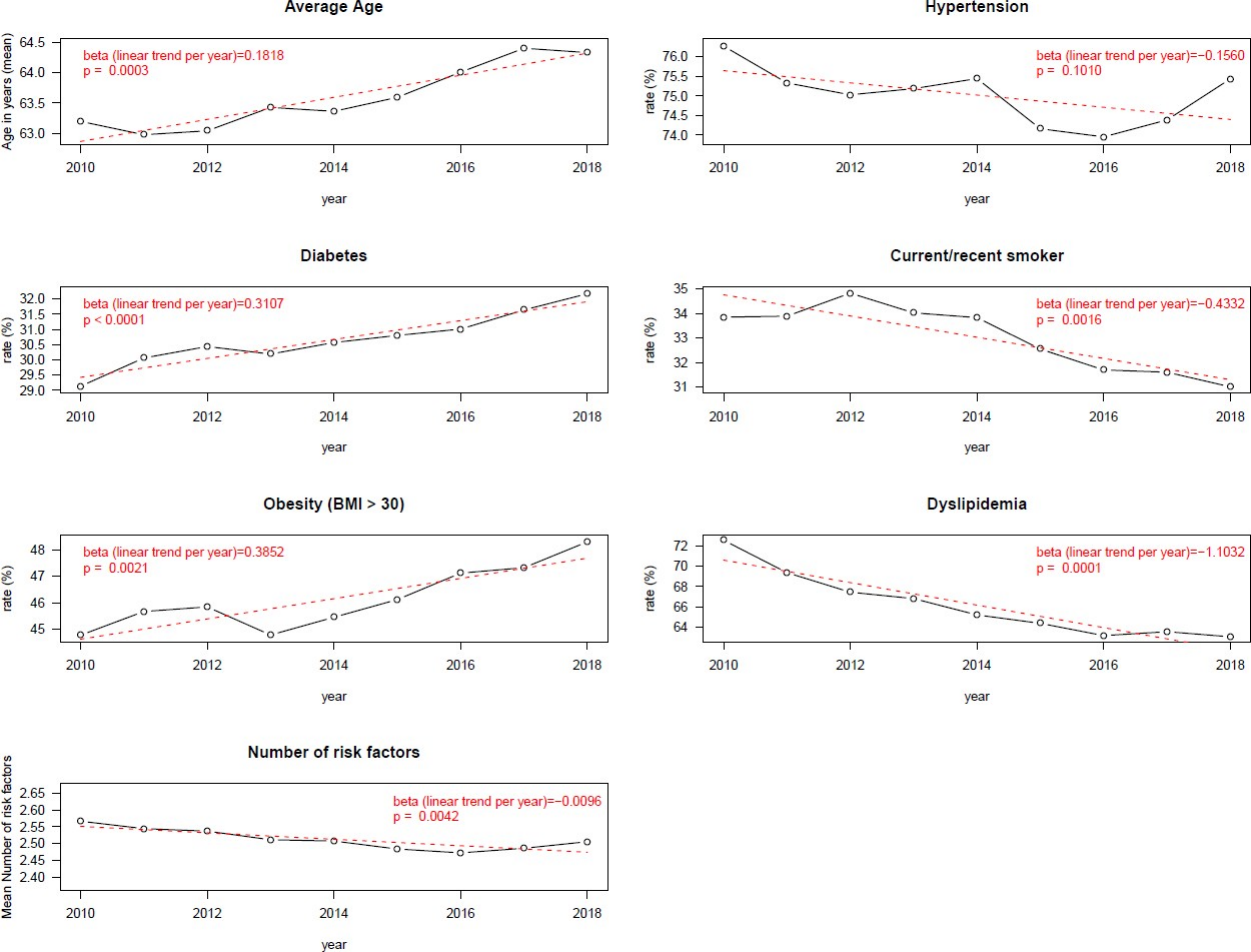
Trends in the prevalence of risk factors over the study period (2010–2018). The linear trend per year (beta coefficient) and its corresponding p-value (red text) are in the upper corner of each graph. The linear trend line is shown as a red dashed line.

The average age of presentation and prevalence of risk factors over the study period are shown in **Fig 3**. From 2010 to 2018, the average age of presentation increased slightly. During this time period the prevalence of diabetes and obesity increased. Conversely, the prevalence of smoking and dyslipidemia decreased. The prevalence of hypertension remained generally consistent. Additionally, the mean number of risk factors remained largely unchanged over the study period.

## Discussion

This study has four principal findings. First, the overwhelming majority of patients with CAD undergoing their first PCI have at least one traditional risk factor, with a majority having 3 or more. Only a very small proportion of patients have none of the traditional risk factors. Second, women generally present at a later age compared with men, except in the small subset of patients who had more than 4 risk factors, where this gap was markedly reduced. Third, since 2010, the prevalence of diabetes and obese patients has increased among patients presenting for their first PCI, whereas the prevalence of smoking and dyslipidemia has decreased. Finally, smoking and obesity appear to have an outsized impact on age at the time of presentation, with smokers presenting over a decade earlier than non-smokers and obese patients presenting approximately 4 years earlier than non-obese patients.

The importance of conventional risk factors of CAD has been established by multiple large cohort studies [10, 11]. For instance, Khot et al examined the prevalence of risk factors for coronary heart disease among patients enrolled in 14 multinational clinical trials in 1990s and early 2000s. They found that most patients had at least one conventional risk factor and the average number of risk factors in their population was 1.3, where the risk factors studied were cigarette smoking, diabetes, dyslipidemia, and hypertension [12]. Additionally, in their study, they recognized that 15.4% of women and 19.4% of men had none of the conventional risk factors [12]. In contrast, we found that the average number of risk factors in our population was 2.5, and only 3.4% of women and 5.1% of men had none of the conventional risk factors. Although this difference may be due to the inclusion of obesity as an independent risk factor in our study, even when Khot et al included obesity as a risk factor the prevalence of patients without risk factors was only reduced to 8.5% in women and 10.7% in men. The proportion of patients with none of the traditional risk factors in our study was smaller than previously reported. Patients with one or less risk factors presented at an earlier age than those with two risk factors, and are thus a population that merits further investigation.

As previously demonstrated, women presented at an older age [11, 13, 14]. As the number of risk factors increased, the difference in age at the time of presentation between men and women narrowed, such that in patients with more than four risk factors the difference between the age of men and women was less than a year. Women are on average 10 years older when they develop CAD, which may be related to differences in sex hormones and differences in age at which risk factors first appear [15]. Further, it is known that total cholesterol levels peak a decade later in women compared with men, which may partially explain the difference in age at the time of presentation [16].

Perhaps the most important finding of our study is that current/recent cigarette smoking—potentially a completely preventable risk factor—continues to be widely prevalent and appears to be associated with almost a decade earlier presentation of CAD. This effect is seen in both men and women and contributes to both AMI and non-AMI presentations. In addition, the association appears to be additive to all of the other traditional risk factors. The importance of reducing smoking rates and possible approaches for doing so are well established [17, 18]. In 2013, it was estimated that a reduction in the smoking rate by 10% in Michigan would be associated with a 3.95% reduction in the total number of PCI procedures performed for patients with STEMI [19].

Similar to smoking, we found obesity to be associated with earlier age at the time of presentation of CAD, which was also seen in both AMI and non-AMI presentations and among both genders. Previous studies have shown that patients with extreme obesity (defined as having a BMI>40 kg/m^2^) experience their first myocardial infarction approximately 12.5 years earlier compared with patients with a BMI between 18.6–25.0 kg/m^2^ [20]. Moreover, our data suggest that the impact of obesity is additive to that of smoking. While smoking prevention has been a difficult problem to solve, the reduction of obesity in the population requires an even more complex and multifaceted solution. Because obesity is related to clinical conditions as well as socioeconomic factors like food access, poverty, and physical activity environment, obesity prevention requires wide-reaching public policy measures [21]. Physicians must work with lawmakers, school districts, and the broader community to lower obesity rates. Our findings call for renewed efforts to focus on smoking cessation and obesity prevention to reduce the impact of CAD.

This study should be interpreted in the context of the following limitations. First, we do not have data on the prevalence of risk factors in the broader community (i.e. patients who did not undergo PCI). Thus, our data cannot be used to estimate the anticipated benefit of public health measures to reduce smoking or obesity prevention. Secondly, our study only evaluated patients who underwent PCI which we used as a proxy for CAD. We did not evaluate patients who were treated with coronary artery bypass surgery or those with CAD who did not undergo revascularization. However, PCI remains the most common treatment modality for revascularization of CAD. Additionally, while it is clear that obesity is a risk factor for cardiovascular disease, it has been shown that defining obesity by waist-to-hip ratio rather than BMI can enable more accurate estimation of risk in most ethnic groups [22]. Waist-to-hip ratio is not recorded in the registry. Lastly, the diagnosis of risk factors was based on physician or patient report and was not independently verified.

## Conclusion

The majority of patients undergoing their first PCI for CAD have two or more coronary risk factors. Smoking and obesity are associated with an earlier age of presentation and appear to be additive. Our findings call for continued efforts aimed at preventing and treating smoking and obesity.

## Abbreviations & acronyms

BMC2: Blue Cross Blue Shield of Michigan Cardiovascular Consortium
CABG: coronary artery bypass graft surgery
CAD: Coronary Artery Disease
MI: myocardial infarction
PCI: Percutaneous Coronary Intervention
RFs: Risk factors
